# Provision of Psychotherapy One Year after the Beginning of the COVID-19 Pandemic in Austria

**DOI:** 10.3390/ijerph18115843

**Published:** 2021-05-29

**Authors:** Elke Humer, Barbara Haid, Wolfgang Schimböck, Andrea Reisinger, Marion Gasser, Heidrun Eichberger-Heckmann, Peter Stippl, Christoph Pieh, Thomas Probst

**Affiliations:** 1 Department for Psychotherapy and Biopsychosocial Health, Danube University Krems, 3500 Krems, Austria; christoph.pieh@donau-uni.ac.at (C.P.); thomas.probst@donau-uni.ac.at (T.P.); 2 Austrian Federal Association for Psychotherapy, 1030 Vienna, Austria; haid@transformberatung.com (B.H.); wolfgang.schimboeck@liwest.at (W.S.); office@reisingerandrea.at (A.R.); gassermarion@hotmail.com (M.G.); peter@stippl.info (P.S.); 3 ABILE-Viktor Frankl Education Austria, 3390 Melk, Austria; 4 PROGES, 4020 Linz, Austria; heidrun.eichberger@progres.at

**Keywords:** psychotherapy, telephone, internet, attitudes, COVID-19

## Abstract

A previous study revealed that the majority of Austrian psychotherapists switched to remote settings during the first months of the COVID-19 pandemic. The current study investigated whether this change in treatment format was maintained after one year of the COVID-19 pandemic. From 16 February until 2 April 2021, a total of 238 Austrian psychotherapists completed an online survey. They were asked about the number of patients currently treated in-person, via telephone and via the internet. Psychotherapists rated three different aspects of psychotherapy (ability to actively listen to patients, ability to understand what is going on in the patients and ability to support patients emotionally) for three different formats (in-person with facemasks, telephone and internet) separately. The results show that, after one year of the pandemic, the majority (78.4%) of patients were treated in-person (compared to 21.7% during the first months of the COVID-19 pandemic; *p* < 0.001). This change in the treatment format was accompanied by a strong increase in the total number of patients treated by 77.2% on average (*p* < 0.001). Psychotherapists reported no differences between in-person psychotherapy with facemasks and psychotherapy via the internet with regard to the three investigated aspects of psychotherapy, while the surveyed aspects were rated less favorably for psychotherapy conducted via telephonic communication (*p* < 0.05). Further studies are needed to investigate the reasons why most psychotherapists switched back to the in-person format with the continuation of the COVID-19 pandemic.

## 1. Introduction

The COVID-19 viral pandemic and its associated stressors have been reported to have a strong impact on the human psyche [1]. A study conducted at the beginning of the COVID-19 pandemic in Austria (April 2020) on a representative sample of the Austrian general population revealed a strong increase in mental health problems compared to pre-pandemic studies. A high prevalence of mental health disorders was observed, such as 21% for depression, 19% for anxiety and 16% for insomnia [2]. About 10 months after the COVID-19 outbreak in Austria, during the second wave of COVID-19 infections (December 2020–January 2021), a further increase in mental health disturbances was observed (26% depression, 23% anxiety, 18% insomnia) [3], indicating an increased need for mental health care during, as well as in the aftermath of, the COVID-19 pandemic.

Before the COVID-19 pandemic, the usual psychotherapeutic format was the in-person setting. Efforts to contain the spreading of the virus mainly rely on the reduction of physical contacts. Therefore, treating patients remotely (i.e., via the internet or telephone) became a valuable option to enable the continuation of psychotherapeutic treatment while adhering to the COVID-19 safety measures [4]. During the first year of the COVID-19 pandemic, several studies conducted around the world reported an increased switch to remote settings at the expense of in-person settings, as reviewed recently [5]. One of the first studies investigating changes in the psychotherapeutic treatment format due to the pandemic was conducted in Austria [6]. In Austria, the first COVID-19 cases were reported at the end of February 2020, which was followed by strict COVID-19 lockdown measures from the middle of March 2020 until the end of April 2020. With the end of the lockdown, daily COVID-19 cases decreased and remained at a low level until the end of June 2020. Thereafter, movement restrictions were reduced and traveling was allowed to countries with low numbers of COVID-19 cases. From July to November 2020, daily COVID-19 cases started to increase again. To combat the rapid spread of the virus during this second wave, the government introduced a second obligatory COVID-19 lockdown from the middle of November until the beginning of December 2020. After a few weeks of relaxed lockdown measures, a third strict lockdown went into effect at the end of December 2020, which ended at the beginning of February 2021. Despite the strict nationwide lockdown measures, daily confirmed cases remained at a high level at the end of the third strict lockdown. As hospitalization rates increased in eastern Austria, the government introduced further strict regional lockdown measures. Thus, the eastern region of Austria went into a fourth strict lockdown, starting during the Easter holidays (April 2021) and lasting until the beginning of May. The strict lockdown measures entailed restrictions in movement and activities with a few exceptions, such as meeting the necessary basic needs of daily life, fulfilling work responsibilities and undertaking outdoor activities alone or with pets or people from the same household. Like other health-care treatments, psychotherapy was one of the few exceptions to the full lockdown restrictions. However, general precautions, such as keeping a safe distance between people, had to be maintained. Although permitted, Austrian psychotherapists strongly reduced in-person psychotherapy sessions during the early weeks of the first COVID-19 lockdown [6]. In brief, in a large survey conducted during the first weeks of the first COVID-19 lockdown in Austria (24 March until 1 April 2020), psychotherapists (*n* = 1547) reported a strong decrease (−81%) in the average number of patients treated in-person per week, whereas the number of patients treated via telephone (+979%) and the internet (+1561%) strongly increased. These changes did not differ between the four therapeutic orientations eligible in Austria (psychodynamic, humanistic, systemic and behavioral). It can be assumed that the main reason for this pronounced change in the treatment format was the aim of reducing the risk of infection for both psychotherapists and patients [6]. Also, the fact that most insurance companies started to reimburse the costs for remote psychotherapy during the first COVID-19 lockdown likely facilitated the provision of remote psychotherapy in Austria [5]. With the continuation of the COVID-19 pandemic, further specific recommendations for non-medical health professionals (including psychotherapists) were provided by the Austrian Federal Ministry of Social Affairs, Health, Care and Consumer Protection [7]. These recommendations included safety measures such as wearing of facemasks, keeping a safe distance of at least 2 m, frequent ventilation and increased cleaning and disinfection in the treatment rooms. The Austrian Federal Association for Psychotherapy recommended practitioners to switch to remote psychotherapy if possible and provided further specific information to their members about safety measures for psychotherapy conducted in-person [8]. We previously reported that being able to adhere to protective measures against COVID-19 significantly reduced the fear of becoming infected with COVID-19 in psychotherapists conducting in-person psychotherapy during the early weeks of the first COVID-19 lockdown in Austria [9].

In a previous survey, Austrian psychotherapists and patients experiencing a change from in-person to remote psychotherapy or vice versa during the COVID-19 pandemic were surveyed about six months after the beginning of the COVID-19 pandemic (during the summer months in 2020). Among the 133 participating patients, 96% experienced changes from in-person to remote psychotherapy and 66% experienced changes from remote to in-person psychotherapy. The 217 participating psychotherapists changed from in-person to remote psychotherapy with, on average, 11.2 (standard deviation (SD) = 10.1) patients and from remote psychotherapy to in-person psychotherapy with, on average, 9.62 (SD = 10.3) patients [10]. However, whether the strong changes in treatment format toward remote psychotherapy observed in Austria at the beginning of the COVID-19 pandemic were maintained one year after the beginning of the pandemic has not been assessed in detail so far. Therefore, research question (RQ) 1 of this study aimed to investigate the format in which psychotherapy was delivered by Austrian psychotherapists after one year of the COVID-19 pandemic and the potential differences compared to the first weeks of the COVID-19 pandemic. We were also interested in whether differences exist concerning the therapeutic orientation of the psychotherapists.

In general, studies highlight that psychotherapists are often more negative about remote psychotherapy settings than their patients [11]. Previous studies have revealed that remote psychotherapy can be as effective as in-person psychotherapy, and even that therapeutic alliance can be as good as in psychotherapies conducted with in-person contact [12,13,14,15,16]. The study conducted among psychotherapists during the first weeks of the COVID-19 pandemic in Austria in April 2020 revealed that psychotherapists judged remote psychotherapy to be better than expected but not fully comparable to in-person psychotherapy. Differences were observed between therapeutic orientations [4]. Another study conducted during the summer of 2020 showed that therapists perceived several therapeutic interventions to be less typical for remote as compared to in-person treatment; however, patients did not experience such large differences in therapeutic interventions between in-person and remote treatment [10]. To gather further information about differences in the therapeutic process between in-person and remote settings, RQ 2 of the current study aimed to investigate potential differences in the ratings of three important psychotherapeutic process variables (ability to actively listen to patients, ability to understand what is going on in the patients and ability to support patients emotionally) concerning three COVID-19 related psychotherapy formats: in-person with facemask, telephone and internet.

## 2. Materials and Methods

An online survey was designed in REDCap (Vanderbilt University, Tennessee, United States) [17,18], comprising 40 items in total. The study was supported by the Austrian Federal Association for Psychotherapy (Vienna, Austria), who informed their members about the study. Additionally, the link to the online survey was sent to all licensed Austrian psychotherapists registered in the list of psychotherapists of the Austrian Federal Ministry of Social Affairs, Health, Care and Consumer Protection who had provided a valid e-mail address (about 6000 psychotherapists).

The survey was open from 16th February 2021 to 2nd April 2021. Participation was voluntary, without incentives. Participants had to agree to the data protection declaration to start the survey (electronic informed consent). The principles outlined in the Declaration of Helsinki were followed and the Ethics Committee of the Danube University Krems (Krems, Austria) approved the study.

One year earlier, during the first COVID-19 lockdown in Austria (i.e., between 24 March and 1 April 2020), an online survey of Austrian psychotherapists was conducted investigating the number of patients treated per psychotherapy format as well as attitudes toward remote psychotherapy. Results from this survey have been published previously [4,6,19] and data on the number of patients treated per treatment format were used in the current study to investigate potential differences in therapy format after one year of the COVID-19 pandemic. Both surveys were cross-sectional and conducted anonymously so no information regarding repeated measures is available, i.e., the 2020 sample could be totally different from the 2021 sample or there could be an overlap.

### 2.1. Measures

The following items were assessed in the current survey, as well as in the survey conducted in 2020. Psychotherapists were asked about their age, gender, level of qualification (i.e., in training under supervision or licensed), how long they had been registered in the official Austrian list of licensed psychotherapists and their therapeutic orientation (i.e., psychodynamic, humanistic, systemic and behavioral). Psychotherapists were asked about the average number of patients currently treated per week per treatment format (in-person, telephone and internet).

The following items were assessed only in the survey conducted in 2021. Psychotherapists were asked whether they had conducted in-person psychotherapy with facemasks. Psychotherapists were further asked to rate the following three different aspects of the therapeutic process on a five-point scale from 1 = ”never” to 5 = “always”: (1) being able to actively listen to patients; (2) being able to understand what is going on in the patients; (3) being able to support patients emotionally. Therapists were asked to rate these three process variables for three different psychotherapy formats (in-person with facemasks, telephone and internet) separately if they answered that they used the respective format.

### 2.2. Statistics

Statistical analyses were performed with SPSS Version 26 (IBM Corporation, Armonk, NY, USA).

Potential differences in sociodemographic characteristics were analyzed by *t*-tests for independent samples and chi-squared tests.

Differences in the numbers of patients treated in total and separated by treatment format in 2021 vs. 2020 were analyzed by independent *t*-tests (dependent *t*-tests were not used as the two surveys were cross-sectional, as noted before). Univariate analyses of variance (ANOVAs) were computed to assess differences with respect to the therapeutic orientation in 2021, only including the therapists who could be assigned to one therapeutic orientation (i.e., the nine psychotherapists who were educated in more than one psychotherapeutic orientation were excluded).

Mixed ANOVAs (RM-ANOVAs) were performed to investigate perceived differences with respect to three different psychotherapeutic process variables (active listening, understanding what is going on in the patients and being able to support patients emotionally) between three different psychotherapy formats (in-person with facemasks, telephone and internet). The rating of how well psychotherapists were able to realize the three different aspects of the therapeutic process was the dependent variable. Initially, we intended to include the therapeutic orientation (four levels: psychodynamic, humanistic, systemic and behavioral) as a between-subject factor. Due to the low number of psychotherapists with experience in treating patients with all three analyzed psychotherapeutic formats (*n* = 64), it was not possible to further differentiate between therapeutic orientations. Therefore, the therapeutic orientation was not included as a between-subject factor in the model. The Greenhouse–Geisser corrected values are presented. Bonferroni corrections were applied for the pairwise post hoc tests.

All statistical analyses were two-tailed, with an alpha level of 0.05 indicating statistical significance.

## 3. Results

### 3.1. Study Sample

In total, *n* = 238 psychotherapists completed the online survey in 2021. Psychotherapists were M = 50.97 (SD = 9.88) years old and 76.9% were female (compared to 73.5% female in the Austrian list of psychotherapists in April 2021). The majority (*n* = 221, 92.9%) of the participants were licensed psychotherapists. Years in the profession since registration in the Austrian list of psychotherapists was 12.10 (SD = 9.70). The distribution of their therapeutic orientation was 16.4% psychodynamic, 50.8% humanistic, 20.2% systemic and 8.8% behavioral. The remaining 3.8% could not be allocated to one orientation (multiple answers were possible). As summarized in Table 1, participating psychotherapists did not differ in gender, age, years in the profession and orientation from those participating in the survey conducted one year earlier (all *p*-values ≥ 0.159). The only difference was that in 2021 17 psychotherapists (7.1% of the total sample) who were not yet registered in the official list of licensed psychotherapists but who were practicing under supervision also participated in the survey, whereas in 2020 this group of psychotherapists was not invited to participate.

### 3.2. Results for RQ1: Provision of Psychotherapy during the Second Year of the COVID-19 Pandemic

A total of six psychotherapists (2.5%) did not provide any in-person psychotherapy in 2021, while in 2020 the majority of the psychotherapists (898, 58%) did not conduct in-person psychotherapy at all.

A closer look at the sample surveyed in 2021 revealed that among those psychotherapists not conducting any in-person psychotherapy, one psychotherapist did not practice at all, three practiced via telephone and the internet and two solely via the internet. In total, 39 (16.4%) psychotherapists only practiced in-person and the majority (193; 81.1%) practiced in-person as well as remotely. A total of 82 therapists used all three therapy formats. The number of psychotherapists who reported having treated patients in-person with facemasks was 189 (79.4%). Of the 49 psychotherapists who never treated patients in-person with facemasks, 44 stated that they were currently treating patients in-person.

The total number of patients (in-person plus telephone plus internet) treated during the first COVID-19 lockdown increased by 77.2% during the second year of the COVID-19 pandemic (*p* < 0.001; Table 2).

An analysis per treatment format revealed that the total number of patients treated in-person increased by 437% (*p* < 0.001), while the number treated via telephone decreased by 64% (*p* < 0.001) and the number treated via the internet decreased by 21% (*p* = 0.017). Table 2 also summarizes the percentages of patients treated per treatment format (the number of patients treated per treatment format was related to the total number of patients treated at the individual level of the psychotherapist). It can be seen that, during the first COVID-19 lockdown in 2020, only 21.7% of the patients were treated in-person, whereas this proportion increased to 78.4% in 2021 (*p* < 0.001). For psychotherapy via telephone, a strong decrease from 48.2% to 8.6% was observed (*p* < 0.001), and the proportion of patients treated via the internet was more than halved (from 30.1% to 13.0%; *p* < 0.001) in the second year of the COVID-19 pandemic in Austria.

Results concerning the effect of therapeutic orientation on the number of patients treated before the COVID-19 pandemic as well as during the first COVID-19 pandemic have been reported previously [6]. As no effect of the therapeutic orientation was observed in the survey conducted in 2020, only data obtained in the current survey were analyzed in the following.

The total number of patients treated did not differ between therapeutic orientations (*p* = 0.087; Table 3). The numbers of patients treated in-person (*p* = 0.781) and via the telephone (*p* = 0.378) also did not differ, whereas a difference was observed for psychotherapy via the internet (*p* = 0.018). Bonferroni-corrected post hoc tests revealed that systemic psychotherapists treated more patients (M = 3.54) via the internet than psychodynamic therapists (M = 1.51; *p* = 0.047). All other pair-wise post hoc tests did not reach significance. When the numbers of patients treated per psychotherapy format were related to the total number of patients treated, no significant differences were observed between psychotherapeutic orientations (*p* ≥ 0.096; data not shown).

### 3.3. Results for RQ 2: Rating of Different Aspects of the Psychotherapy Process per Treatment Format

In total, 64 psychotherapists stated that they were currently using three different formats (in-person with facemasks, telephone and internet).

Results of the RM-ANOVAs revealed an overall difference in the rating for whether psychotherapists could actively listen to their patients with regard to treatment format (*p* = 0.036; Table 4). Pairwise Bonferroni-corrected post hoc tests revealed a higher/better rating for psychotherapy via the internet compared to psychotherapy via telephone (*p* = 0.011), whereas no difference was observed between in-person psychotherapy with facemasks and psychotherapy via the telephone (*p* = 0.171), nor between in-person psychotherapy with facemasks and psychotherapy via the internet (*p* = 1.000).

Therapists reported differences in the ability to understand what is going on in their patients with regard to the format in which psychotherapy was provided (*p* < 0.001). Treating patients via the internet ((*p* < 0.001) or in-person with facemasks (*p* = 0.005) was rated as enabling a better ability to understand what is going on in the patients compared to psychotherapy via telephone.

The rating of whether psychotherapists could support their patients emotionally differed between the treatment formats (*p* < 0.001). Pairwise Bonferroni-corrected post hoc tests revealed a higher rating for in-person psychotherapy with facemasks (*p* = 0.001) and psychotherapy via the internet (*p* < 0.001) compared to psychotherapy via telephone.

## 4. Discussion

This research aimed to investigate the formats in which psychotherapy was delivered by Austrian psychotherapists after one year of the COVID-19 pandemic and to elucidate potential differences compared to the first weeks of the COVID-19 pandemic. We further aimed to investigate psychotherapists’ ratings of their ability to actively listen to patients, understand what is going on in patients and emotionally support patients with respect to the format in which the psychotherapy was provided.

One major finding was that after one year of the pandemic, the total number of patients treated increased by on average 77.2% compared to the first COVID-19 lockdown. After one year of the pandemic, the majority (78.4%) of patients were treated in-person, while during the first COVID-19 lockdown the patients treated in-person were a minority (21.7%) [6]. During the first COVID-19 lockdown, the preferred format for remote psychotherapy was the telephone, whereas psychotherapy via the internet was more common than psychotherapy via the telephone after one year of the pandemic. Consistent with our earlier study, no relevant differences in the number of patients treated per treatment format were observed between the four therapeutic orientations eligible in Austria [6]. The results suggest that with the prolongation of the COVID-19 pandemic most psychotherapists switched back to in-person psychotherapy. Moreover, it seems that those psychotherapists treating remotely tended to move to more advanced treatment formats, such as videoconferencing as compared to telephonic communication. While during the first COVID-19 lockdown an undersupply of psychotherapy was suggested [6], it seems that the average number of patients treated after one year of the pandemic (M = 17.93, SD = 9.35) exceeded pre-pandemic levels (M = 14.04, SD = 11.32 [6]) by 27.7%. This is in line with the strong increase in mental health issues observed in the Austrian general population compared to pre-pandemic data [2,3,20], suggesting an increased demand for psychotherapeutic support.

A further major finding was that psychotherapists reported no differences in their ability to actively listen to their patients, understand what is going on in their patients or support patients emotionally whether patients were treated in-person with facemasks or via the internet. All three important psychotherapeutic process variables were rated less favorably for psychotherapy conducted via telephonic communication. Consistent with our previous study, psychotherapists rated psychotherapy via the internet more comparably to in-person psychotherapy than to psychotherapy via telephone [4]. However, previous research has revealed that remote sessions are experienced as being more superficial than in-person sessions [21] and specific therapeutic interventions have also been rated to be more typical in in-person settings as compared to remote settings by therapists [10]. Thus, aspects other than the three investigated variables likely contributed to the increased utilization of in-person psychotherapy compared to psychotherapy via the internet. We hypothesize that common drawbacks of remote psychotherapy (i.e., the perceptions of impersonality due to the lack of physical presence, technological problems, extra effort or hassle [11]) might have contributed to the trend towards decreased utilization of remote psychotherapy formats with the continuation of the COVID-19 pandemic. However, it might also have been that in-person psychotherapy was mainly conducted without facemasks, as the number of patients treated in-person was not further differentiated with regard to safety measures.

This study has several limitations. First, it was not possible to merge data of both surveys at the individual level, as no personal data to identify individuals was collected due to data protection reasons. Although no differences in the sociodemographic characteristics of the participants of the two surveys were observed, potential differences between the responders of the two surveys concerning the preferred treatment format cannot be excluded. As the study was conducted online, a respondent bias toward higher participation of psychotherapists with a higher preference for psychotherapy via the internet is possible. Thus, the results might not be generalizable to the general psychotherapeutic situation in Austria and it is possible that the results even overestimate the proportion of patients being treated remotely after one year of the COVID-19 pandemic in Austria. A further shortcoming is that all ratings took only the psychotherapists’ perspective into account. Additionally, the items to assess the variables were self-constructed, they were one-item answers (to keep the survey short to reduce drop-outs), and no psychometrically sound instruments were used. As there are large differences between countries with respect to health intervention policies, the results might not be generalizable to other countries with long traditions of remote psychotherapy or different health care systems.

## 5. Conclusions

In conclusion, with the increased duration of the COVID-19 pandemic, psychotherapists treated the majority of their patients in the conventional in-person setting. Although psychotherapy via the internet was rated similarly to psychotherapy conducted in-person with facemasks and the study was conducted during a time when several lockdown measures were in place, the majority of patients were treated in-person. Further studies are needed to investigate the underlying reasons why most psychotherapists switched back to the in-person setting with the continuation of the COVID-19 pandemic in Austria. The results suggest a need for the implementation of mental health intervention policies to cope with the increased demand in psychotherapeutic support during and in the aftermath of the COVID-19 pandemic efficiently and effectively. Future studies should also elucidate whether this observed trend towards decreased utilization of remote psychotherapy formats with the continuation of the COVID-19 pandemic also takes place in other countries.

## Figures and Tables

**Table 1:** Sociodemographic characteristics of the samples. Tests for independent samples were used as both surveys were cross-sectional.

Characteristics	2020	2021	Statistics
**Gender ^1^**			
Female, *n* (%)	1171 (75.7)	183 (76.9)	X^2^ (1) = 0.260;
Male, *n* (%)	376 (24.3)	54 (22.7)	*p* = 0.610
Diverse, *n* (%)	0 (0)	1 (0.4)	
			
**Age in years,** M (SD)	51.67 (9.69)	50.97 (9.88)	T (1783) = 1.034;
			*p* = 0.301
**Qualification**			
In training under supervision, *n* (%)	0 (0)	17 (7.1)	X^2^(1) = 111.562;
Licensed, *n* (%)	1547 (100)	221 (92.9)	*p* < 0.001
			
**Years in profession ^2^,** M (SD)	11.19 (9.20)	12.10 (9.70)	T(1755) = −1.409;
			*p* = 0.159
**Orientation ^3^**			
Psychodynamic, *n* (%)	324 (21.2)	39 (17.0)	X^2^(3) = 3.432;
Humanistic, *n* (%)	716 (46.8)	121 (52.8)	*p* = 0.330
Systemic, *n* (%)	340 (22.2)	48 (21.0)	
Behavioral, *n* (%)	151 (9.9)	21 (9.2)	

^1^ Only male and female psychotherapists were included in the statistical analysis. ^2^ Set to “0” for all psychotherapists in training under supervision. ^3^ Only psychotherapists who could be classified under one orientation were included (16 psychotherapists participating in the survey in 2020 and 9 psychotherapists participating in the survey in 2021 were excluded).

**Table 2:** Numbers of patients treated on average per week in 2020 vs. 2021 for the three treatment formats.

Variable	2020, M (SD) *n* = 1547	2021, M (SD) *n* = 238	Statistics
Total	10.12	17.93	T (1309.3) = −12.335; *p* < 0.001
	(9.05)	(9.35)	
In-person	2.60	13.97	T (1264.1) = −30.949; *p* < 0.001
	(4.75)	(7.89)	
Telephone	4.53	1.61	T (1655.1) = 12.95; *p* < 0.001
	(5.77)	(2.65)	
Internet	2.99	2.36	T (1351.8) = 2.406; *p* = 0.017
	(4.44)	(3.69)	
In-person, %	21.70	78.36	T (1367.2) = −33.707; *p* < 0.001
	(28.72)	(23.11)	
Telephone, %	48.15	8.62	T (1914.4) = 31.76; *p* < 0.001
	(34.78)	(12.97)	
Internet, %	30.14	13.02	T (1485.4) = 11.519; *p* < 0.001
	(31.66)	(18.95)	

**Table 3:** Numbers of patients treated during the COVID-19 lockdown per treatment format and orientation.

Variable	Orientation	M (SD)	Statistics
Total	Psychodynamic	16.67 (8.05)	F (3;225) = 2.217; *p* = 0.087
	*n* = 39		
	Humanistic	17.66 (8.43)	
	*n* = 121		
	Systemic	20.44 (10.53)	
	*n* = 48		
	Behavioral	15.24 (8.69)	
	*n* = 21		
In-person	Psychodynamic	13.36 (7.90)	F(3;225) = 0.361; *p* = 0.781
	*n* = 39		
	Humanistic	13.93 (7.17)	
	*n* = 121		
	Systemic	14.79 (8.96)	
	*n* = 48		
	Behavioral	13.05 (7.51)	
	*n* = 21		
Telephone	Psychodynamic	1.79 (2.64)	F(3;225) = 1.036; *p* = 0.378
	*n* = 39		
	Humanistic	1.46 (2.56)	
	*n* = 121		
	Systemic	2.10 (3.32)	
	*n* = 48		
	Behavioral	1.05 (1.53)	
	*n* = 21		
Internet	Psychodynamic	1.51 (2.16)	F(3;225) = 3.429; *p* = 0.018
	*n* = 39		
	Humanistic	2.27 (3.32)	
	*n* = 121		
	Systemic	3.54 (5.09)	
	*n* = 48		
	Behavioral	1.14 (1.53)	
	*n* = 21		

**Table 4:** Ratings of different aspects of the psychotherapy process per treatment format (*n* = 64).

Aspect	M	SD	Statistics
Active listening			
In-person with facemasks	4.63	0.519	F (2; 63) = 4005.880; *p* = 0.036
Telephone	4.44	0.639	
Internet	4.64	0.55	
Understanding what is going on in patients			F (2; 63) = 3711.902; *p* < 0.001
In-person with facemasks	4.20	0.694	
Telephone	3.87	0.766	
Internet	4.34	0.623	
Being able to support emotionally			F (2; 63) = 3076.131; *p* < 0.001
In-person with facemasks	4.25	0.713	
Telephone	3.91	0.729	
Internet	4.23	0.684	

## Data Availability

The raw data supporting the conclusion of this article will be made available by the authors upon reasonable request.
